# Digital Addiction and Occupational Health in Medical Residency

**DOI:** 10.1192/j.eurpsy.2026.10826

**Published:** 2026-07-31

**Authors:** N. Rmadi, R. Affes, A. Kchaou, F. Dhouib, M. Hajjaji, K. Jmal Hammami

**Affiliations:** 1 Occupational medicine department, Hedi Chaker university hospital; 2 Family medecine department, Faculty of medecine of Sfax, Sfax, Tunisia

## Abstract

**Introduction:**

Digital addiction among medical residents may compromise both professional efficiency and psychological well-being. Understanding its occupational health implications is essential for designing preventive strategies.

**Objectives:**

To assess the occupational health implications of digital addiction among medical residents and examine its impact on work performance and psychosocial well-being.

**Methods:**

A nationwide cross-sectional survey was conducted in 2025 among 429 residents from Tunisia’s four medical faculties. Data included personality traits (BFI-20), smartphone and social media addiction (SAS-SV, BSMAS), and work performance (IWPQ). Associations and mediation models were analyzed.

**Results:**

Conscientiousness was the strongest predictor of high work performance (β=0.488, p<0.001). However, its protective role was significantly attenuated by social media addiction (indirect effect β=–0.141, p=0.002). Nearly half of residents were addicted to smartphones and one in four to social media, with addiction linked to lower performance and higher counterproductive behaviors.

**Conclusions:**

Digital addiction represents an emerging occupational health risk in residency training. Preventive interventions—such as digital hygiene workshops, smartphone-free clinical environments, and psychosocial support—are needed to protect residents’ performance and well-being. Integrating these measures into occupational health policies could help sustain efficiency and patient safety.

**Disclosure of Interest:**

None Declared

